# Solvent-Induced Supramolecular Assembly of a Peptide-Tetrathiophene-Peptide Conjugate

**DOI:** 10.3389/fchem.2019.00467

**Published:** 2019-06-28

**Authors:** Zongxia Guo, Yujiao Wang, Xiao Zhang, Ruiying Gong, Youbing Mu, Xiaobo Wan

**Affiliations:** ^1^College of Chemistry and Molecular Engineering, Qingdao University of Science and Technology, Qingdao, China; ^2^Key Laboratory of Biobased Polymer Materials, Shandong Provincial Education Department, College of Polymer Science and Engineering, Qingdao University of Science and Technology, Qingdao, China; ^3^Qingdao Institute of Bioenergy and Bioprocess Technology, Chinese Academy of Sciences, Qingdao, China; ^4^Key Laboratory of Optoelectronic Chemical Materials and Devices, School of Chemical and Environmental Engineering, Jianghan University, Ministry of Education, Wuhan, China

**Keywords:** oligopeptide, oligothiophene, peptide-tetrathiophene-peptide, supramolecular assembly, supramolecular chirality

## Abstract

The assembly of a peptide-tetrathiophene-peptide (PTP) conjugate has been investigated in mixed solvents, which has different polarities by changing the solvent proportions. It was found that PTP can form fibers in THF/hexane solutions with 40–80%v of hexane. The fibers were stable and did not change on time. On the other hand, PTP formed ordered structures in a mixed solution with the water content from 40 to 60%v. For the as-prepared solutions, two nanostructures vesicles and parallelogram sheets were obtained. The parallelogram sheets could transform into vesicles on time. The fibers showed supramolecular chirality, however, there was no Cotton effect for vesicles and parallelogram sheets. UV-vis, FL, XRD, FT-IR, and CD spectra together with SEM, AFM, TEM were used to characterize the nanostructures and properties of the assemblies. Molecular packing mechanism was proposed based on the experimental data.

## Introduction

The performance of the functional materials based on these systems is largely affected by the nanostructures of the assemblies, therefore the manipulation of the nanostructures is one of the most important issue to tune their properties (Ajayaghosh et al., 2006; Zhang et al., 2009; George et al., 2012; Mitra et al., 2013; Shin et al., 2013; Baram et al., 2014; Liu et al., 2014). Especially, different nanostructures with distinct properties can be constructed from the same entity by adjusting the outside environment. Many strategies, such as guest induction (Janssen et al., 2010), light (Diegelmann et al., 2012; Samanta et al., 2012), pH (Mba et al., 2011; Draper et al., 2017), and solvent polarity (Hu et al., 2012; Guo et al., 2017a), have been used to assemble various superstructures based on the biomolecule-assisted self-assembly of π-conjugated systems.

The chiral property endows the materials with potential applications, such as chiral sensing, chiral switch, and asymmetric catalysis etc., (Zhang et al., 2014; Kim et al., 2015; Jiang et al., 2016; Jia et al., 2019). Supramolecular chirality is highly related to the packing mode of molecules, thus different packing styles may lead to diverse supramolecular chirality even when the chirality at the molecular level is the same. So, controlling the chiral properties of self-assemblies based on the biomolecule-π-conjugated systems is an important issue. Bäuerle et al. have tuned the supramolecular chirality of the assembled oligothiophene nanostructures by decorating carbohydrate or amino acid with different chirality (Schmid et al., 2009; Digennaro et al., 2013; Schillinger et al., 2013). Wei and et al. reported a sugar-based amphiphilic perylene diimide derivative (PTCDI-HAG) which self-assembled into two kinds of fibers with opposite supramolecular chirality by using binary solvents (Huang et al., 2011). Yao and Zhan et al. showed that the chirality of nanohelices formed from a tripeptide-perylene diimide (PDI) conjugate could be reversed through heating and ultrasound treatment (Ke et al., 2013). In these previous reports, although the supramolecular chirality could be tuned, no distinct nanostructure change was observed, for example, fibers are still fibers although its twisting direction changed. On the other hand, distinct nanostructure change which is accompanied by supramolecular chirality change might provide alternative ways for chirality sensing materials. However, inspecting the relationship between distinct assembled nanostructure (Echue et al., 2015) and its supramolecular chirality is rarely reported especially based on the self-assembly of π-conjugated systems assisted by biomolecules (Shang et al., 2017).

**Figure 1:**
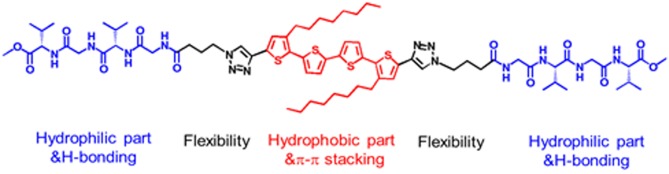
Chemical structure of PTP.

Here, a peptide-tetrathiophene-peptide (PTP) conjugate (Figure 1) was used as a model object to explore the manipulation of morphology and supramolecular chirality of the assemblies via simply changing the solvent polarity. There are some significant features for PTP: Gly-(L-Val)-Gly-(L-Val) segments at both ends of the thiophene backbone are relatively hydrophilic than the thiophene core and can provide hydrogen bond sites through which β-sheet structures could be formed (Wall et al., 2011; Guo et al., 2013); the thiophene core decorated with two octyls in the middle is a hydrophobic part and can form π-π stacking as well (Lehrman et al., 2012; Guo et al., 2014); the linkers between the thiophene and peptide are flexible and then the spatial location of both hydrophilic and hydrophobic parts in the supramolecular assemblies can be freely tuned under certain conditions. Thus PTP gives chances to tune the self-assembly by verifying the solvent polarity, since H-bonding and π-π stacking are the driving forces for the self-assembly of peptide-thiophene conjugates (Guo et al., 2017b).

## Results and Discussions

The synthesis of PTP has been reported in our previous report (Guo et al., 2013). And it could form gels in some aprotic solvents (THF, dioxane, and acetone) and also protic solvents (methanol and ethanol) as reported in our previous work. Among these solvents, the critical gel formation concentration in THF is the highest, indicating the better solubility. In the present investigation, the assembly of PTP in solution were performed in pure THF, or mixed solvent composed of THF at very low concentration. The impact of different proportions of the mixed solvent on the nanostructures and properties were studied. Moreover, concentrations and time effect were also checked.

### Solvent Induced Formation of Nanostructures

Firstly, the self-assembly of PTP was performed in THF with the concentration at 0.1 mg/mL (6.61 × 10^−5^ M). It was found that no well-organized nanostructures can be formed in THF solution Figure S1). Then the mixed solvent was used to detect if the nanostructures change when varying the solvent polarity. The solvent with high polarity (water) or low polarity (hexane) was added to THF to enhance or decrease the polarity of the mixed solvent. The proportions of the mixed solvent could be varied to obtain solvents with different polarities. For the preparation of the samples, PTP was dissolved in THF to make a parent solution and then water or hexane was added to get the solution with different volume ratio of the two solvents. The samples were stirred vigorously for 10 min and then kept at room temperature for a certain period of time.

**Figure 2:**
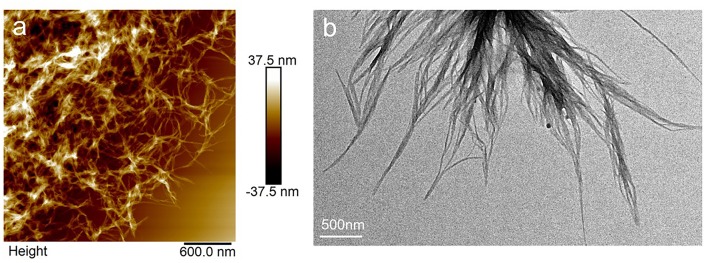
AFM **(a)** and TEM **(b)** images of assembled structures of PTP in THF/hexane (1:1, v).

For PTP in THF/hexane solution with a concentration at 0.1 mg/mL, it was observed that ordered nanostructures could be formed only in a certain range of hexane content (40–80%v). The morphology of the assembled PTP in THF/hexane (40–80%v) was always fiber structures (Figure S2). Figure 2 shows the nanostructures in AFM and TEM images formed from the THF/hexane solution. It is clear that fibers were formed in the solvent with 50%v of hexane. PTP assembled into fibers with widths of 43 ± 15 nm and heights of 13 ± 6 nm (Figures 2a,b; Figure S3). The width of fibers was measured as 32 ± 17 nm. The difference for the above data might be due to the expansion effect from the AFM tip and the possible drying effects on substrate. Inspecting the fibers carefully, thick ones were formed from thin ones as can be seen also in the SEM images (Figure S2). It should be noted that no obvious chiral structures could be observed indicating that the chirality from the peptide did not transfer into the assembled nanostructures (Yang et al., 2013; Wang et al., 2018), although supramolecular chirality might be formed in the assemblies. This formation of fiber in THF/hexane was also confirmed by SEM characterization (Figure S2). Aging treatment had no impact on such fiber structures.

**Figure 3:**
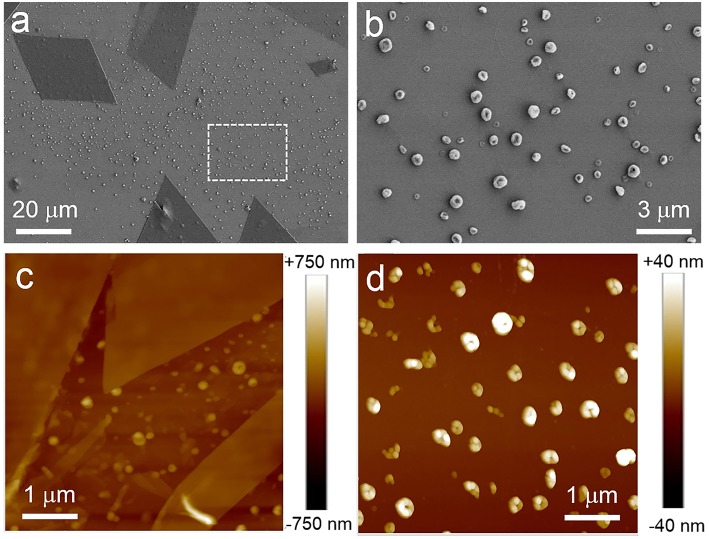
SEM **(a,b)** and AFM **(c,d)** images of the as-prepared assembled structures of PTP in THF/water solution. **(b)** was zoomed in from the area enclosed by white dotted line in **(a)**. **(a,c)** showed the nanostructures composed of sheet structures and spherical ones. **(b,d)** indicated the spherical structures.

It was the other case when mixed solvent composed of water was used. It was found that ordered structures can be formed in a narrow range of water content (40–60%v), and two nanostructures always could be formed in such range of water content for the as-prepared solutions. Figure 3 showed SEM and AFM images obtained from THF/water solutions (water, 50%v). It is obvious that sheet and spherical structures were formed. Some sheets were parallelograms. The thickness of the sheets ranges from tens to hundreds of nanometers (Figure S4).

**Figure 4:**
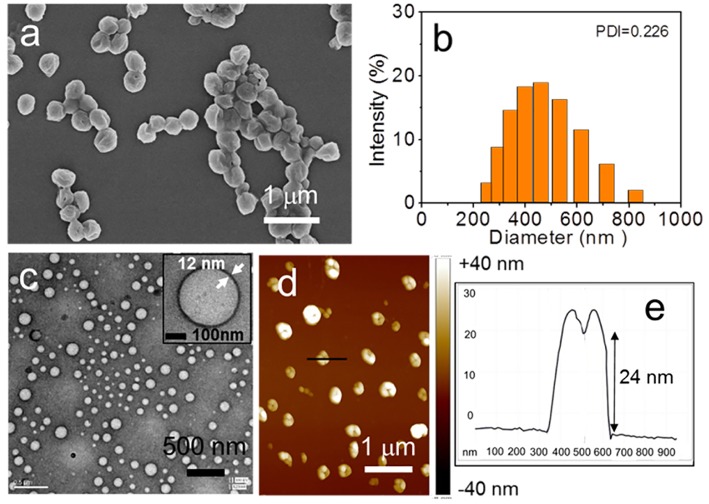
The assembled structures of PTP in THF/water with 4 days of aging: **(a)** SEM image, **(c)** TEM image, and **(d)** AFM image. **(b)** Size distribution measurement of the vesicles by DLS. The section profile of one vesicle in **(d)** was shown in **(e)**.

It should be noted that there is a time-dependent transformation of the nanostructures form sheet to spheres. As time went on, sheets structures gradually became into spheres. After 4 days of aging, only spherical aggregates were observed and their morphology was characterized by SEM, TEM, and AFM (Figures 4a,c,d; Figure S5). The diameter of the spheres ranges from 255 to 825 nm as evidenced by Dynamic Light Scattering (DLS) (Figure 4b). Furthermore, such sphere seems sunken from the SEM image, indicating that hollow spheres might be formed. This speculation was proved by TEM measurement, which clearly showed that the wall of sphere, as shown in the inset of Figure 4c. In addition, the morphology in AFM image is very similar to that obtained in the SEM image. Concave spheres were observed (Figure 4d). The section profile of a sphere shows that the ratio of horizontal distance (630–340 = 290 nm) to vertical distance (24 nm) is about 12. This high width to height ratio combined with the observation from SEM, TEM, and AFM images confirmed that vesicles were formed. The thickness of the vesicle wall was estimated to be about 12 nm by TEM image (Figure 4c). From AFM analysis, the thickness of double walls was measured as 22 ± 3 nm in accordance with the TEM results. A typical section profile of a vesicle was shown in Figure 4e.

The concentration impact on the nanostructures was detected as well for the two different mixed solvents. For the assembly of PTP in THF/Hexane (40–80%v), there was no obvious effect of the concentration on the fiber structures. However, it is the other case for the assembly in THF/water (40–80%v). On increasing the concentration of PTP to 0.2 mg/mL (THF/water, 1:1, v), the sheets became the major structures and the vesicles was almost disappeared for the as-prepared solutions (Figure S6). As discussed above, there is time-dependent transformation tendency from sheets to spheres. It is the same case for the high concentration solution. The sheets would transform into vesicles gradually. After 2 days of aging, vesicles appeared (Figure S7). And sheet structures can hardly be found for this solution after 4 days of aging, and only the vesicles showed up (Figure S8). Actually, it was found that the much-diluted solutions had the same tendency to from vesicles after long time of aging, although the number of vesicles was much less than that obtained from the high solutions. Based on the above investigation, it can be concluded that the sheets are dynamic controlled intermediate structures and the vesicles are thermodynamic stable ones.

### Spectral Investigation of the Assembled Nanostructures

#### UV-Vis and FL Spectra

**Figure 5:**
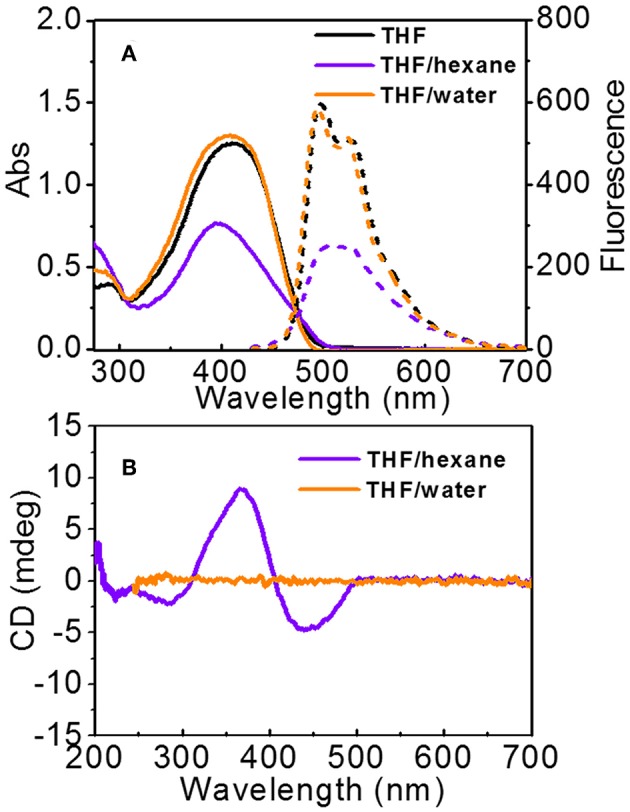
**(A)** UV-vis (solid line) and FL spectra (dashed lines) of PTP in THF, THF/hexane (1:1, v), and THF/water (1:1, v). The excitation is 412 nm for all solutions. **(B)** CD spectra of PTP in THF/hexane and THF/water. [PTP] = 0.1 mg\mL.

UV-vis and FL spectra were used to detect the aggregation of PTP (Figure 5). In THF, PTPs are in the molecularly dissolved state, the maximum absorption of PTP is at 412 nm which belongs to the π-π^*^ transition of the tetrathiophene backbone (Schillinger et al., 2009; Kumar et al., 2011). Two resolved emission peaks of PTP are observed at 498 and 529 nm, which again indicated the molecularly dissolved state of PTPs in THF. In the mixed solvents, absorption/emission band changes occurred and varied according to the polarity of the mixed solvents. In THF/hexane, the absorption was at 398 nm, showing a 14 nm blue-shift compared to that in the molecularly dissolved state, and the absorption intensity decreased apparently. In FL spectra, the emission peaks became unresolved, and moreover, the intensity decreased by 50% with respect to that in THF. Based on the UV-vis and FL data, it was suggested that the PTP formed H-like aggregates in THF/hexane (Wall et al., 2011; Digennaro et al., 2013). While in THF/water, it is clear that the absorption spectrum is very close to that in THF, both in shape and intensity. The maximum absorption is at 409 nm, only a 3 nm of blue shift from that in the molecularly state (412 nm) was observed. The emission of PTP in THF/water was almost the same to that in THF. Only a very small blue shift occurred in accordance with the UV-vis spectra. It could be concluded that the interaction between thiophene backbones was weak and the chromophores were loosely packed in THF/water solution. Overall, spectroscopic data have proved that changing the solvent polarity could induce distinct aggregation modes of the molecules.

#### CD Spectra

Normally, the conjugates of peptide and thiophene will assemble into nanostructures with supramolecular chirality in thiophene parts if the peptide is chiral (Stone et al., 2009; Kumar et al., 2011; Lehrman et al., 2012). Thus, we explored the supramolecular chirality of the assembled vesicles and fibers (Figure 5B). The solution with fibers shows an obvious negative Cotton effect with a negative absorption at 442 nm, a positive absorption at 367 nm, and a crossover at 405 nm. A weak negative band at 284 nm was also found. Since the absorptions at about 400 and 280 nm were assigned to the tetrathiophene backbone, the CD signals should be due to the exciton coupling between thiophene chromophores (Cao et al., 2013). The small difference in the absorption positions in UV-vis and CD spectra would be due to the light scattering (Ajayaghosh et al., 2006). The CD spectrum was inconclusive about the presence of β-sheets in samples. Since molecularly PTP shows no chirality in the thiophene part (Figure S9), it indicated that the chirality of peptide was transferred to thiophene stacks which adopted a helical stacking way, in other words supramolecular chirality was formed consequently. Such helical stacks hierarchically assembled into fiber structures. However, the solution composed of vesicles was surprisingly CD silent from 270 to 700 nm. Only noise signal could be observed in the range from 200 to 250 nm. It was hardly to deduce the formation of regular β-sheets from the CD spectrum. It was indicated that the chirality transfer from peptide to thiophene was prohibited in THF/water solution. Based on the morphology and CD data, we can conclude that the different packing modes of PTP molecules in THF/hexane and THF/water lead to different chirality transfer behavior.

The above investigations were mainly focused on the packing of the thiophene core. FT-IR spectra were used to characterize the assembly of peptide segments (Figure S10). FT-IR data shows that the assemblies of the peptide segments in both cases are similar. Amide I and Amide II were found at 1,635 and 1,540 cm^−1^ in THF/hexane, and they were at 1,635 and 1,549 cm^−1^ in THF/water. Such absorptions indicate that β-sheets between peptide segments were formed in both cases (Diegelmann et al., 2008; Ke et al., 2013). The absorption at 3,289 cm^−1^ from the N-H stretching vibration further confirmed the formation of H-bonds for PTP in both solutions (Lehrman et al., 2012).

**Figure 6:**
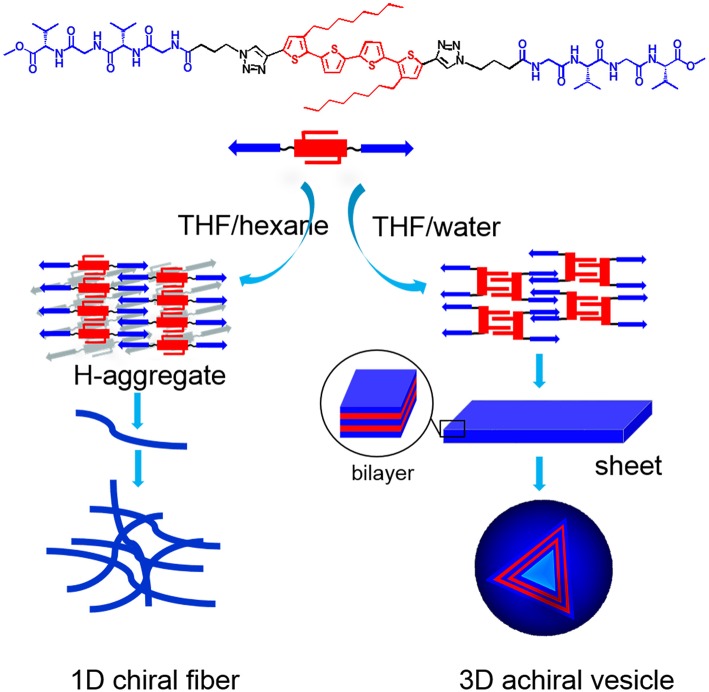
Proposed self-assembly of PTP in different solvents.

Based on the experimental data, molecular packing modes of PTP in THF/hexane and THF/water were proposed (Figure 6). In THF/hexane, it is speculated that the main driving force of self-assembly are hydrogen bond and π-π stacking, through which PTP assembled into H-like aggregates, and such H-like aggregates further hierarchically assembled into thin fibers. These thin fibers could further interact and twist with each other to form thicker ones. During such process of assembly, the chirality of peptide transferred to thiophene units, and helical thiophene stacks were formed. The chirality of peptide finally was expressed into the supramolecular chirality of the assembled nanostructures. On the other hand, since the polarity of the THF/water solution was much more polar than that of the THF/hexane solution, the hydrophobic part of PTP, that is the tetrathiophene core, tends to stay away from the polar solvent. Consequently, we postulated that the flexible linker between thiophene and peptide allows PTP to adopt a conformation different from that in THF/hexane: two octyl chains stay on one side and the two peptide segments stay on the opposite side with the tetrthiophene in the middle. Based on such conformation, octyl chains interdigitate with octyl chains via van de Waals interactions, and peptide segments interdigitate with peptide segments from different PTPs via the hydrogen bonds, and finally 3D vesicles were formed. It is suggested that there are four PTP layers in the normal direction of the vesicle wall. It was estimated that such alignment will give a wall thickness of about 11 nm (Figure S11), which is in good agreement with the AFM and TEM data (12 nm). XRD was also used to check the molecular packing (Figure S12). XRD data of vesicles showed an obvious peak with *d* spacing of 12.7 and 6.2 nm for the vesicles, confirmed the bilayer signature. For the fibers, there was no such peaks. The small peaks with *d* spacing of 0.78 nm were both observed for vesicles and fibers should be from the π-π packing (0.78/2 = 0.39 nm). So, in THF/water, octyl chain-octyl chain interactions and H-bonding interactions between the peptide segments would play an important role in the formation of vesicles. It is interesting that the chirality transfer was blocked from peptide to thiophene in the vesicles. Two reasons might lead to such result in THF/water: (i) the chiral peptide segments prefer to stay on one side of the thiophene and in such conformation the peptide segment are somehow perpendicular to the long axis of thiophene, which might lead to the low possibility of chirality transfer; and (ii) UV-vis and FL spectral data showed very small changes in the aggregation state of PTP in THF/water compared to its dissolved state in THF, indicating that the interactions between thiophene units were not strong enough to generate exciton coupling between chromophores. Based on the above aspects, the chirality transfer was prohibited in the vesicles and supramolecular chirality formation did not occur either. It is clearly evidenced by the present investigation that there is a direct relationship between the assembled nanostructures and the formation of supramolecular chirality.

## Conclusions

In summary, the self-assembly of a PTP conjugate in different solvents was systematically investigated. It reveals that the solvent polarity can induce different ways of hierarchical assembly, leading to different nanostructures and properties, especially the supramolecular chirality. In THF/hexane, chirality transfer from peptide to thiophene occurred and chiral fibers were formed. While in THF/water, vesicles were obtained and chirality transfer was prevented. This investigation highlighted that simple strategy could be used to manipulate the assembled nanostructures. Especially, supramolecular chirality can be also tuned by forming different structures. The present strategy provides opportunities to fabricate assemblies with distinguished supramolecular chirality through manipulating the assembled nanostructures, therefore leading to diverse chiral functionalities, such as sensors or switches.

## Data Availability

No datasets were generated or analyzed for this study.

## Author Contributions

ZG and XW contributed to the idea of the work and the preparation of the manuscript. ZG, YW, XZ, RG, and YM have done all experiments on the assembly and corresponding analysis.

### Conflict of Interest Statement

The authors declare that the research was conducted in the absence of any commercial or financial relationships that could be construed as a potential conflict of interest.
