# Supplementary material for: Solvent-Induced Supramolecular Assembly of a Peptide-Tetrathiophene-Peptide Conjugate
Source: Front Chem. 2019 Jun 28;7:467. doi: 10.3389/fchem.2019.00467 (PMC6611225; doi:10.3389/fchem.2019.00467)
Supplement: Supplementary file 1 [file Data_Sheet_1.pdf]

frontiers

Supplementary Material

Solvent-Induced Supramolecular Assembly of A Peptide-Tetrathiophene-Peptide Conjugate

Zongxia Guo[2*], Yujiao Wang[3], Xiao Zhang[3], Ruiying Gong[4], Youbing Mu[1] and Xiaobo Wan[1*]

[1]Key Laboratory of Optoelectronic Chemical Materials and Devices, Ministry of Education, School of Chemical & Environmental Engineering, Jianghan University, Wuhan 430056, P. R. China, [2]College of Chemistry and Molecular Engineering, Qingdao University of Science and Technology, Qingdao 266042, P. R. China, [3]Key Laboratory of Biobased Polymer Materials, Shandong Provincial Education Department, College of Polymer Science and Engineering, Qingdao University of Science and Technology, Qingdao 266042, P. R. China, [4]Qingdao Institute of Bioenergy and Bioprocess Technology, Chinese Academy of Sciences, Qingdao 266101, China

***Correspondence:**

Zongxia Guo: zxguo@qust.edu.cn; Xiaobo Wan: wanxb@jhun.edu.cn

Sample preparation

The typical sample preparation method is as followed: First, PTP was dissolved in THF to make a parent solution with a certain concentration. For example, addition of 10 mL THF to a 20 mL glass bottle with 2 mg powders to get a parent solution of 0.2mg/mL. Then such parent solution was added with water or hexane according to calculated volume. The concentrations of the obtained solutions were 0.1, 0.02, or 0.2 mg/mL to check the concentration effect. Normally, the concentration was 0.1 mg/mL. The samples were both stirred vigorously for 10 min and then kept the samples for aging. Solutions for repeated trials were always used right after they were prepared. All experiments were performed at 20-25 ℃.

Characterizations

UV-vis and FL spectra were recorded in a quartz cuvet with optical path of 10mm on a Varian Cary 50 UV-Vis spectrophotometer and a HitachiF-4600 Fluorescence spectrophotometer, respectively. CD spectra were measured with a JASCO J-815 spectrophotometer in a quartz cuvet with optical path of 10mm. FT-IR spectra were performed on a Nicolet 6700 FT-IR spectrometer (Thermo Electron Corporation). Samples for FT-IR spectra were casted on silicon wafer (100) and dried in vacuum for 3 days at room temperature. The samples for FT-IR were also for XRD measurement. XRD was performed on Bruker d8 advance X-ray diffractometer with Cu Kα radiation. Samples for AFM and SEM characterizations were casted onto freshly cleaved mica surface, evaporating the solvent at atmosphere naturally first and then drying in vacuum overnight. Before SEM characterization, a thin layer of gold was sputtered onto the samples. SEM measurements were performed on Hitachi S-4800 with an accelerating voltage of 3.0 kV. TEM measurements were performed on Hitachi H-7650. The solutions were casted on Cu grid without staining and dried at room temperature before the TEM observation. AFM observation was performed on a Bruker Dimension Fastscan atomic force microscope. The samples for SEM were also for AFM observation. It should be noted that there might be possible drying effects in the micrographs and also for the FTIR and WXRD characterizations. Dynamic light scattering (DLS) was measured on a ZetasizerNano S90 (Malvern).

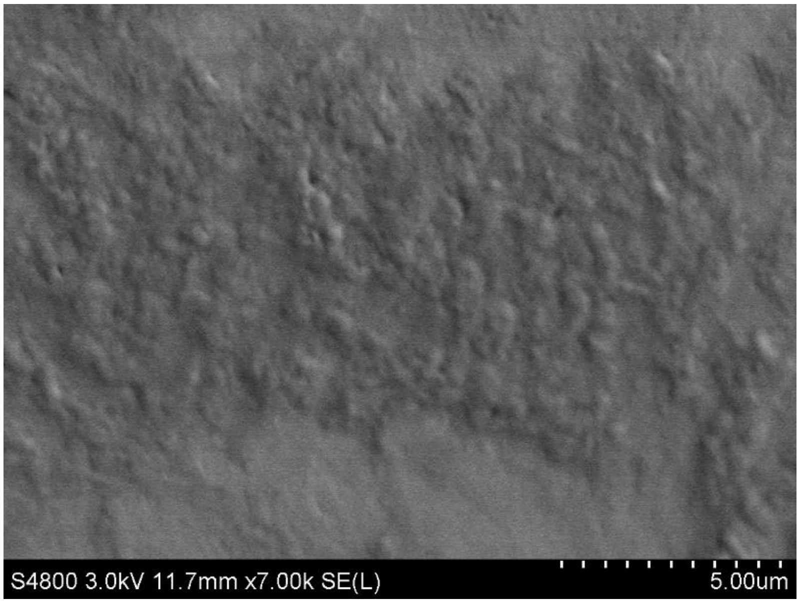

Figure S1 | SEM image of PTP in THF solution. [PTP]=0.1 mg/mL.

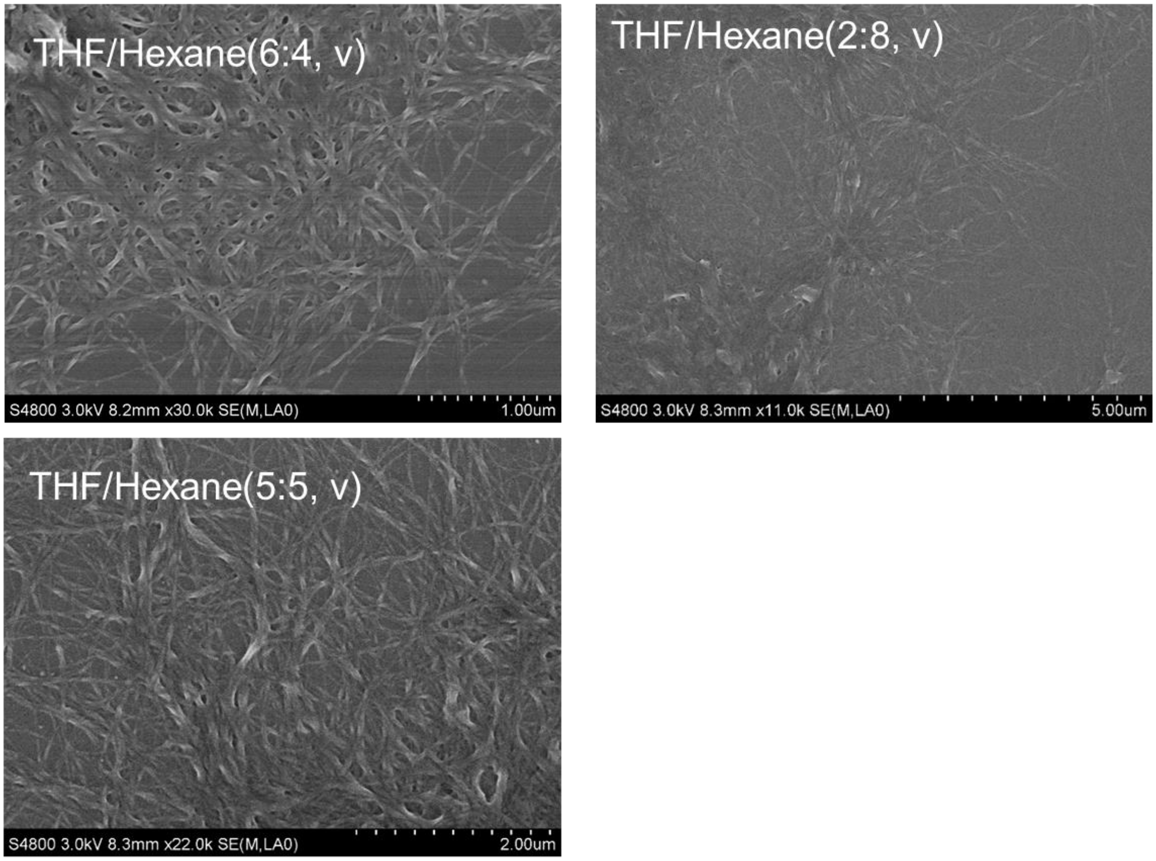

Figure S2 | SEM images of PTP in THF/hexane (1:1, v). [PTP]=0.1 mg/mL.

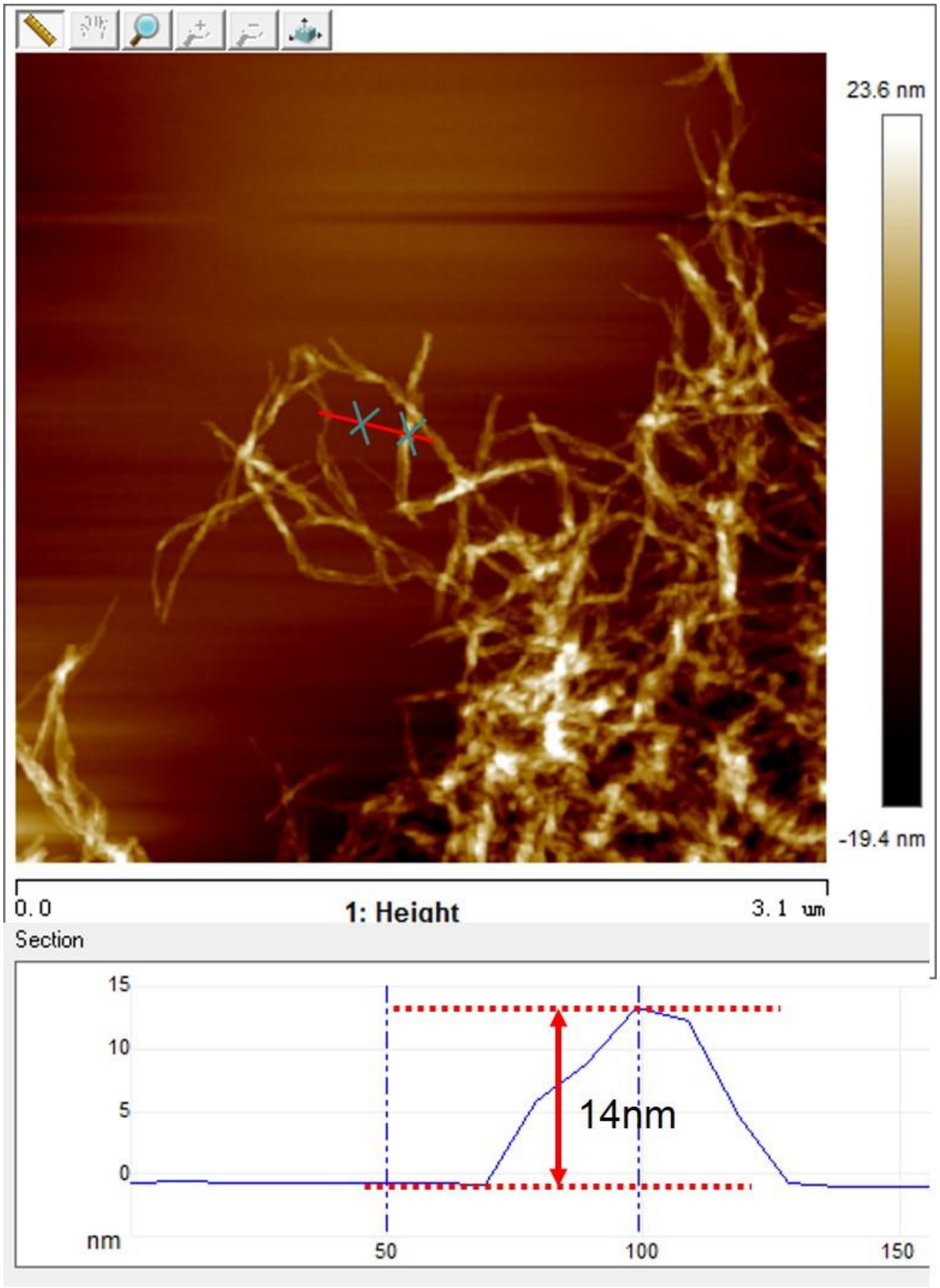

Figure S3 | AFM image of fibers assembled from PTP in THF/hexane (1:1, v) and a section profile. [PTP]=0.1 mg/mL.

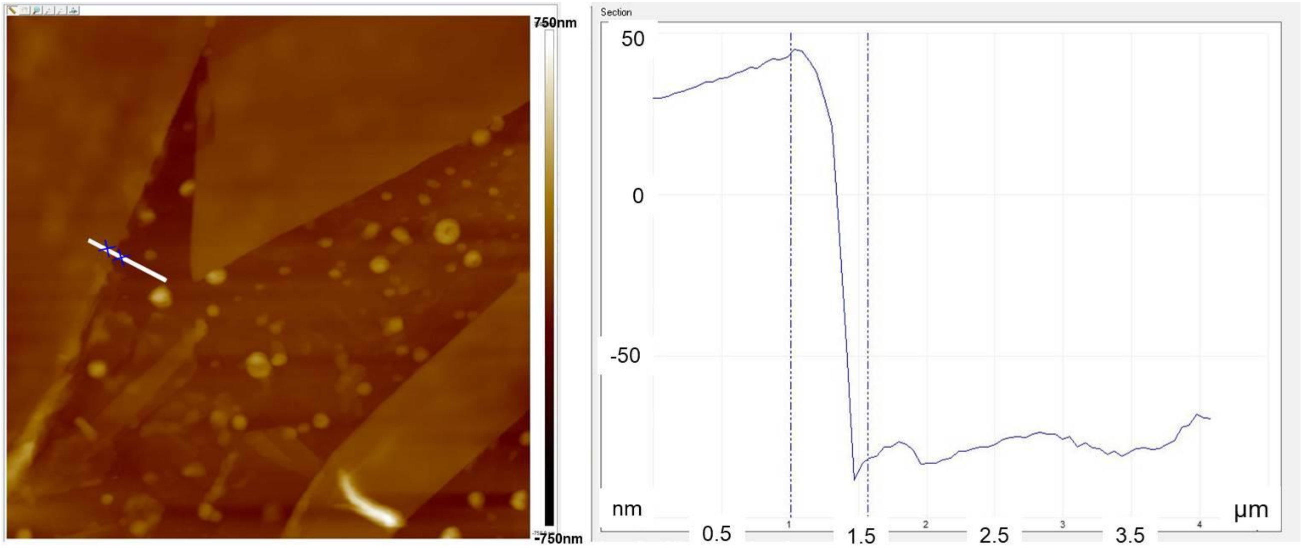

Figure S4 | AFM image and the section file of the as-prepared assembled structures of PTP in THF/water (1:1, v). [PTP]=0.1 mg/mL.

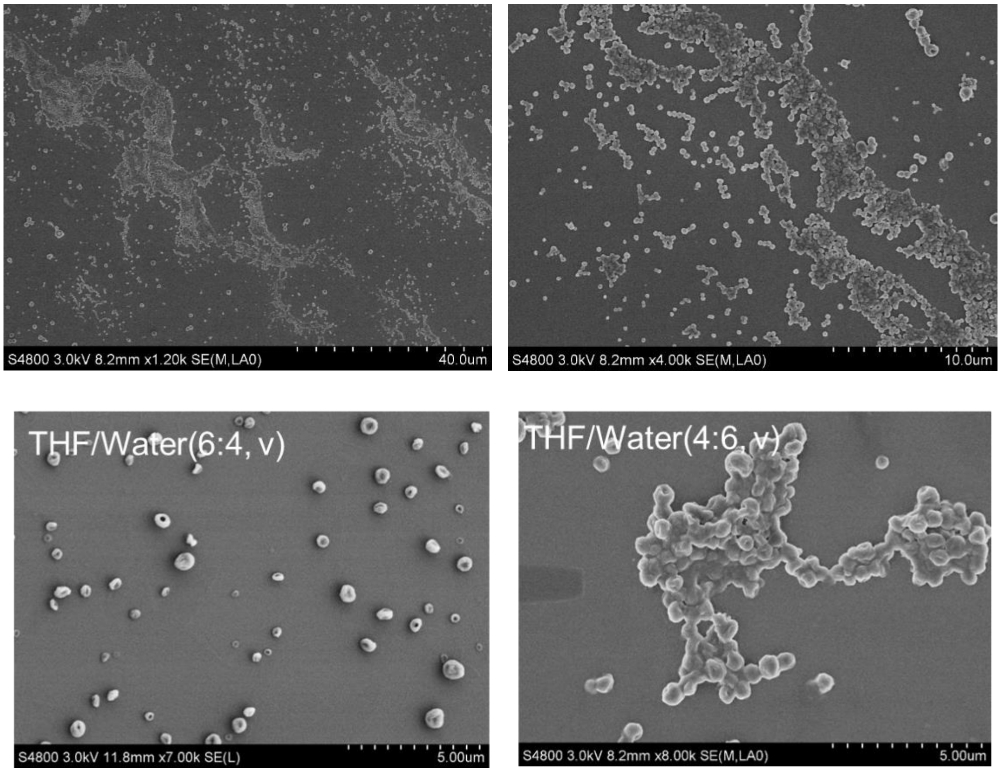

Figure S5 | SEM images of the assembled structures of PTP in THF/water with 4 days of aging. Top two images: THF/water (1:1, v). [PTP]=0.1 mg/mL.

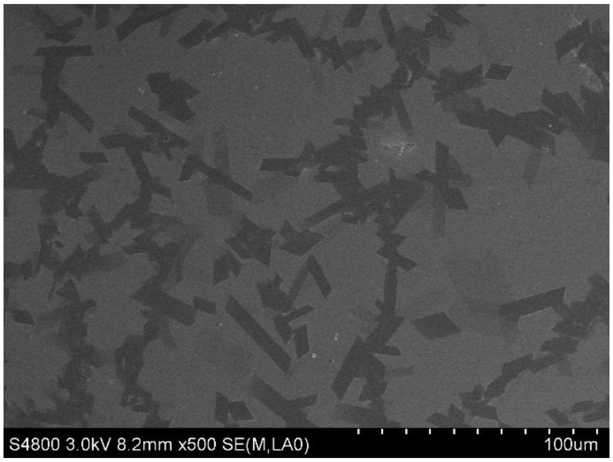

Figure S6 | SEM images of the as-prepared assembled structures of PTP in THF/water (1:1, v). [PTP]=0.2 mg/mL.

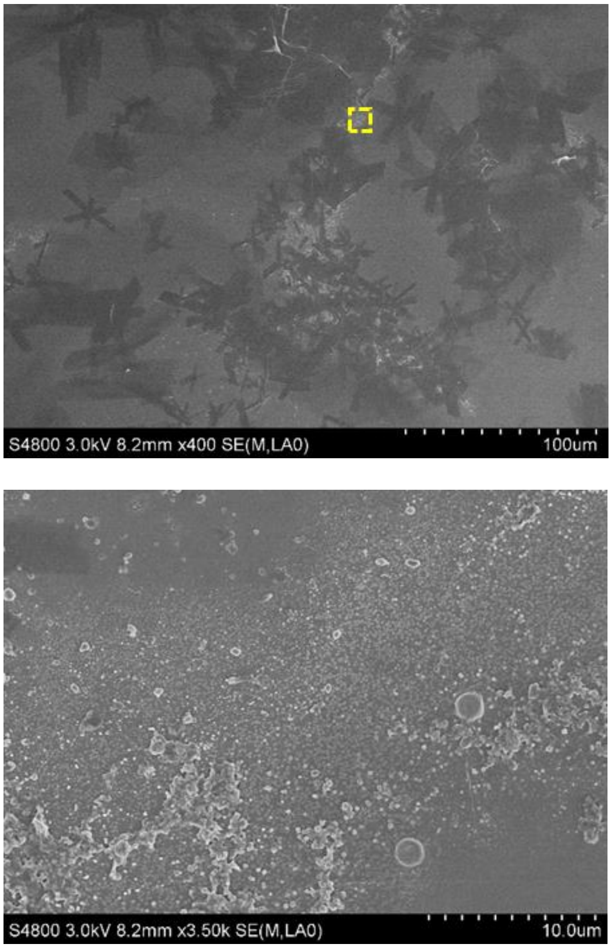

Figure S7 | SEM images of assembled structures of PTP in THF/water (1:1, v) from the solution with 2 days of aging. The bottom image was zoomed in from the top image enclosed by the dotted yellow square. [PTP]=0.2 mg/mL.

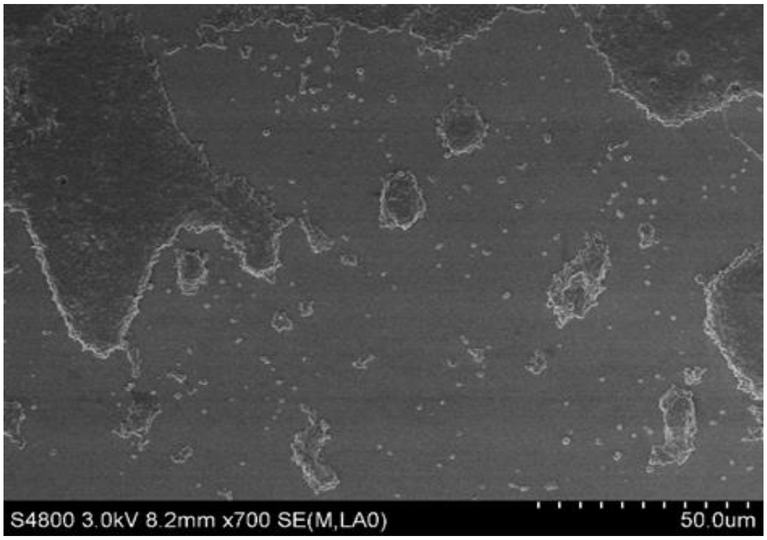

Figure S8 | SEM images of assembled structures of PTP in THF/water (1:1, v) from the solution with 4 days of aging. [PTP]=0.2 mg/mL.

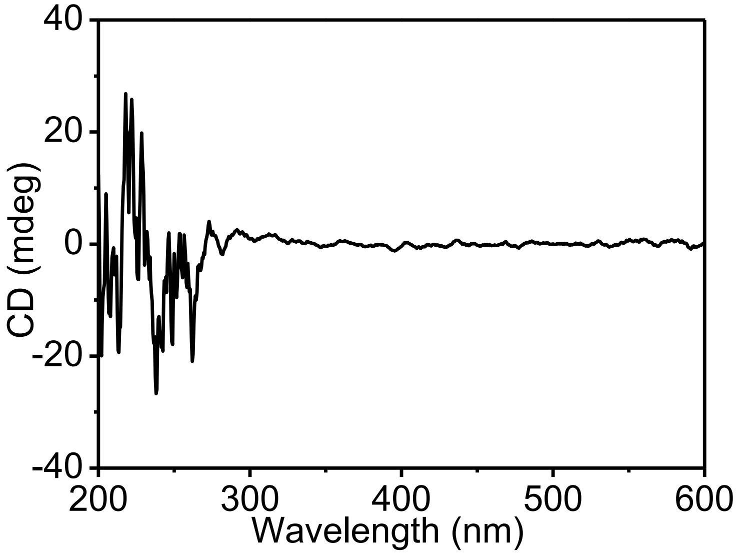

Figure S9 | CD spectrum of PTP in THF. [PTP]=0.1 mg/mL.

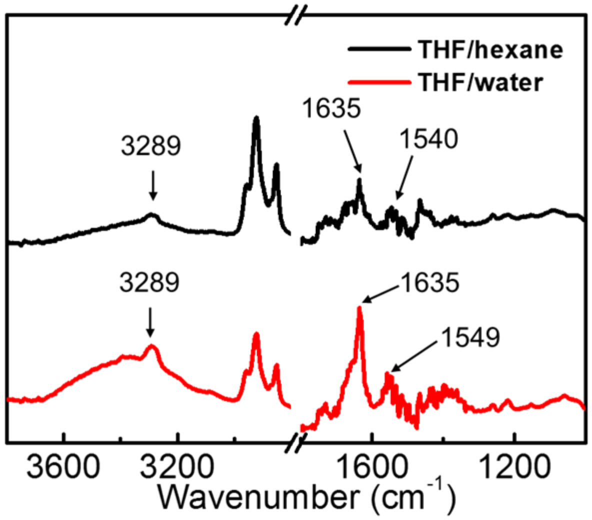

Figure S10 | FT-IR spectra of PTP in THF/hexane (1/1, v/v) and THF/water (1/1, v/v). [PTP]=0.1 mg/mL.

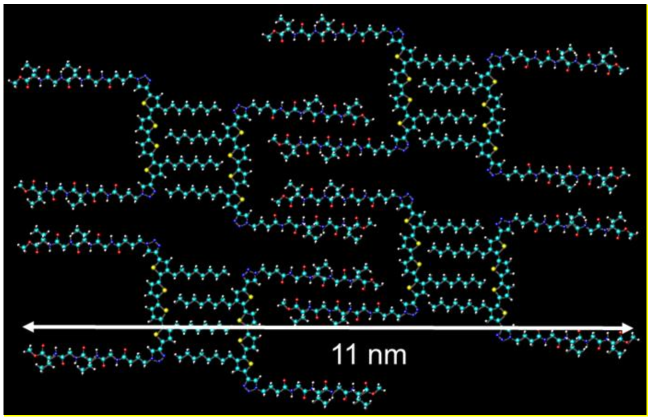

Figure S11 | PTP Packing in vesicle wall. The estimated thickness of the vesicle wall is about 11 nm by the HyperChem software.

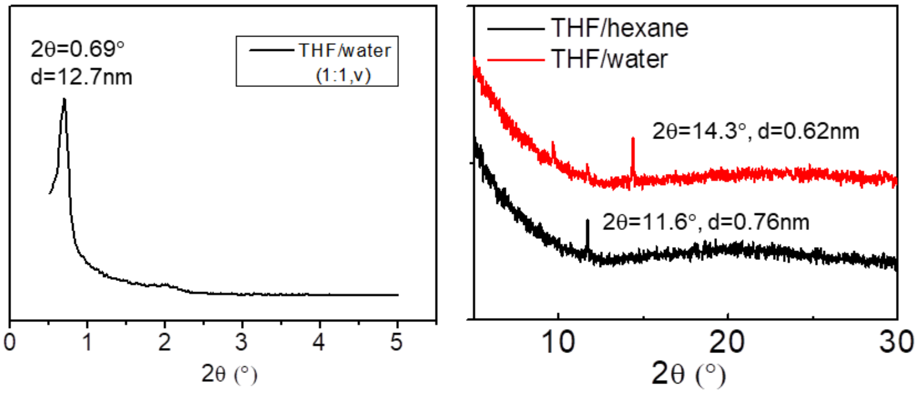

Figure S12 | XRD data of fibers and vesicles.
